# Adherence of pregnant women to Nordic dietary guidelines in relation to postpartum weight retention: results from the Norwegian Mother and Child Cohort Study

**DOI:** 10.1186/1471-2458-14-75

**Published:** 2014-01-24

**Authors:** Anne von Ruesten, Anne Lise Brantsæter, Margaretha Haugen, Helle Margrete Meltzer, Kirsten Mehlig, Anna Winkvist, Lauren Lissner

**Affiliations:** 1 Department of Public Health and Community Medicine, University of Gothenburg, Gothenburg, Sweden; 2 Division of Environmental Medicine, Norwegian Institute of Public Health, Oslo, Norway; 3 Department of Internal Medicine and Clinical Nutrition, University of Gothenburg, Gothenburg, Sweden

**Keywords:** Moba, The Norwegian Mother and Child Cohort Study, Diet, Dietary guidelines, Pregnancy, Postpartum weight retention

## Abstract

**Background:**

Pregnancy is a major life event for women and often connected with changes in diet and lifestyle and natural gestational weight gain. However, excessive weight gain during pregnancy may lead to postpartum weight retention and add to the burden of increasing obesity prevalence. Therefore, it is of interest to examine whether adherence to nutrient recommendations or food-based guidelines is associated with postpartum weight retention 6 months after birth.

**Methods:**

This analysis is based on data from the Norwegian Mother and Child Cohort Study (MoBa) conducted by the Norwegian Institute of Public Health. Diet during the first 4-5 months of pregnancy was assessed by a food-frequency questionnaire and maternal weight before pregnancy as well as in the postpartum period was assessed by questionnaires. Two Healthy Eating Index (HEI) scores were applied to measure compliance with either the official Norwegian food-based guidelines (HEI-NFG) or the Nordic Nutrition Recommendations (HEI-NNR) during pregnancy. The considered outcome, i.e. weight retention 6 months after birth, was modelled in two ways: continuously (in kg) and categorically (risk of substantial postpartum weight retention, i.e. ≥ 5% gain to pre-pregnancy weight). Associations between the HEI-NFG and HEI-NNR score with postpartum weight retention on the continuous scale were estimated by linear regression models. Relationships of both HEI scores with the categorical outcome variable were evaluated using logistic regression.

**Results:**

In the continuous model without adjustment for gestational weight gain (GWG), the HEI-NFG score but not the HEI-NNR score was inversely related to postpartum weight retention. However, after additional adjustment for GWG as potential intermediate the HEI-NFG score was marginally inversely and the HEI-NNR score was inversely associated with postpartum weight retention. In the categorical model, both HEI scores were inversely related with risk of substantial postpartum weight retention, independent of adjustment for GWG.

**Conclusions:**

Higher adherence to either the official Norwegian food guidelines or possibly also to Nordic Nutrition Recommendations during pregnancy appears to be associated with lower postpartum weight retention.

## Background

Pregnancy is a major life event for a woman that together with the natural gestational weight gain (GWG) is often accompanied by changes in diet and lifestyle. Excessive weight gain during pregnancy, however, is also found to be related not only with a higher risk of childhood overweight [1] but also with increased weight retention of the mother in the postpartum period [2-4]. Excessive GWG may thus add to higher risks of developing overweight or obesity in the long term, especially in women with high pre-pregnancy BMI [5-7]. In addition to a higher risk of adverse health outcomes in later pregnancies [5], it is well known that obesity is a risk factor for developing major chronic diseases, such as type 2 diabetes, cardiovascular diseases, or certain cancers [8]. Therefore, prevention of excessive GWG and substantial postpartum weight retention through diet and lifestyle modification during pregnancy is critical. Pregnant women also tend to be more receptive to advice about health behaviour on behalf of their unborn child’s health and furthermore generally desire to get back into shape after delivery.

Although the number of interventional trials to manage weight gain during [9-11] and after [10] pregnancy through a modification of physical activity or diet [9,11,12] is increasing, it has not yet been examined how adherence to specific dietary guidelines during pregnancy affects GWG or postpartum weight. Furthermore, there is uncertainty which specific components of the diet, e.g. nutrients or foods, might have the strongest potential to contribute to prevention of excessive weight gain [13]. Prospective cohort studies investigating effects of specific dietary behaviours during gestation on postpartum weight are generally very scarce. An analysis by Martins *et al.*[14] suggests that increased intake of saturated fat or processed foods during pregnancy is associated with increased weight retention after delivery. A Swedish study investigating the role of meal patterns showed that weight retention 1 year after birth was higher in women with increased frequency of snacking or decreased frequency of lunch eating during or after the pregnancy [15]. Still, the role of specific dietary patterns, such as compliance with national dietary guidelines, during pregnancy on postpartum weight development needs to be elucidated.

Therefore, in the present analysis we used data from the Norwegian Mother and Child Cohort Study (MoBa) to investigate whether adherence to either the official Norwegian food-based guidelines issued in 2011 or the Nordic Nutrition Recommendations issued in 2004, which are nutrient-based, during pregnancy is associated with weight retention 6 months after birth. Furthermore, we studied the relationship between adherence to these dietary guidelines and GWG as a potential intermediate outcome.

## Methods

### Study design and population

The Norwegian Mother and Child Cohort Study (MoBa) is a large prospective population-based pregnancy cohort study conducted by the Norwegian Institute of Public Health [16]. Participants were recruited from all over Norway between 1999 and 2008 by postal invitation after they signed up for a routine ultrasound examination in their local hospital. In total, 40.6% of the invited women were willing to participate in MoBa. The cohort now includes 114,500 children, 95,200 mothers and 75,200 fathers. The participants were asked to provide blood samples and to fill out questionnaires. Follow-up is conducted by questionnaires at regular intervals until the child is 7 years old, and through linkage to national health registries.

Informed consent was obtained from each MoBa participant upon recruitment. The study was approved by The Regional Committee for Medical Research Ethics in South-Eastern Norway.

This study is based on version 5 of the quality-assured data files made available for research in 2010. We considered women with singleton births who have participated in MoBa with not more than one pregnancy and have reported their body weight at the relevant time points (pre-pregnancy, end of pregnancy, and 6 months postpartum). Women with missing FFQ data and an energy intake < 4.5 MJ/day or > 20 MJ/day were excluded [17]. Furthermore, we excluded women with a gestational weight gain or gestational weight loss of > 50 kg, a pre-pregnancy weight of < 35 and > 200 kg, a height of < 1.4 m, a pregnancy duration of < 30 weeks or > 42 weeks as well as those who have delivered a baby of < 600 g.

Accordingly, 47 011 women were eligible for the present analyses.

### Dietary assessment

Habitual diet during pregnancy was assessed with a validated semi-quantitative food-frequency questionnaire (FFQ), which was applied in mid-pregnancy (answered in week 22 of pregnancy). The MoBa-FFQ (http://www.fhi.no/dokumenter/253304bd64.pdf) included 340 questions on the frequency of intake of 255 food items, covering the diet during the first 4-5 months of pregnancy, and is described in detail elsewhere [17].

The FFQ has been thoroughly validated with regard to nutrients, foods, and energy intake within a MoBa sub-sample [18]. Specifically, the intake estimates derived from the FFQ were compared to the following reference measures: a 4-day weighed food diary (FD), a motion sensor (ActiReg®) for measuring total energy expenditure, one 24-h urine collection for analysis of dietary biomarkers including urinary flavonoids, nitrogen and iodine excretion, and a venous blood specimen for analysis of dietary biomarkers including plasma carotenoids, 25-hydroxy-vitamin D, and serum folate. The average correlation coefficient between the FFQ and FD for daily intake was satisfactory and was higher for foods (Spearman’s r = 0.48) than for nutrients (Spearman’s r = 0.36). Furthermore, a cross-classification of estimated intakes from the FFQ and FD showed that the majority (68%) of the participants were classified into the same or adjacent quintiles. Under-reporting of energy intake was more pronounced with the FD than with the FFQ as indicated by comparison to the objectively measured total energy intake. Finally, the FFQ was also able to distinguish between high and low intakes of fruits and vegetables [19], vitamin D, folate, protein, and iodine as confirmed by the biological markers [18-20].

### Diet adherence scores

#### HEI-NFG score

The degree of adherence to the official Norwegian food guidelines (NFG), which are published by the Norwegian health directorate (see Table 1), was measured by means of a diet adherence index, mainly based on food groups. The NFG incorporates quantitative recommendations on the following dietary components: fresh fruit, vegetables, whole-grain, fish, fatty fish, red meat, salt, and added sugar. The corresponding food items of the FFQ were assigned to the respective food groups (see Additional file 1: Table S1) and mixed dishes were disaggregated into their ingredients to improve the estimation of intake of vegetables, fish, and red meat. The recommended intake for salt was transferred to sodium (using the conversion factor 2.5) since in MoBa data sodium rather than salt intake was estimated. The MoBa FFQ did not ask about the use of table salt, therefore the estimated intake of sodium only mirrors the consumption of processed and convenient food.

**Table 1:** **Overview of Norwegian food guidelines and Nordic Nutrition Recommendations**^**1**^

**Norwegian Food Guidelines (NFG) **[21]^**1**^	
**Food group**	**Recommended intake**
Fresh fruits [22]	Min. 300 g/day
Vegetables [22]	Min. 300-450 g/day
Whole-grain	Min. 75 g/day per 10 MJ (2400 kcal) = ca. 70 g/day for women
Fish	300-450 g/week
Fatty fish	Min. 200 (to max. 450) g/week
Red meat	Max. 500 g/week
Processed meat	Limit consumption
Salt	Max. 6 g salt/day ( = 2.4 g sodium/day)
Added sugar	Max. 10% of total energy intake
**Nordic Nutrition Recommendation (NNR) **[23,24]^**2**^	
**Macronutrient**	**Recommended intake**
Total fat	25-35 E%
Saturated + *trans* fat	Not more than 10 E%
Monounsaturated fat	10-*20* E%
Polyunsaturated fat	5-10 E%
Fibre	*At least* 25-35 g/day
*Added* Sugar	Not more than 10 E%
Total protein	10-20 E%

E%: percentage of total energy intake.

^**1**^Nordic Nutrition Recommendations are relevant for all Nordic countries whereas the food-based recommendations just refers to Norway.

^2^5^th^ edition of NNR [24] was considered to include some updates (printed in italics).

Inspired by the US-Healthy Eating Index [25,26], we calculated the diet adherence score, denoted as Healthy Eating Index (HEI)-NFG, by scoring the ratio of the reported and recommended intake of these food groups included into the NFG. Thereby, a higher compliance with each of the single NFG recommendations was reflected by higher score values and the possible score for each component ranged from 0 (non-consumption of the respective food group) to 10 (perfect compliance with recommended intake).

**Figure 1:**
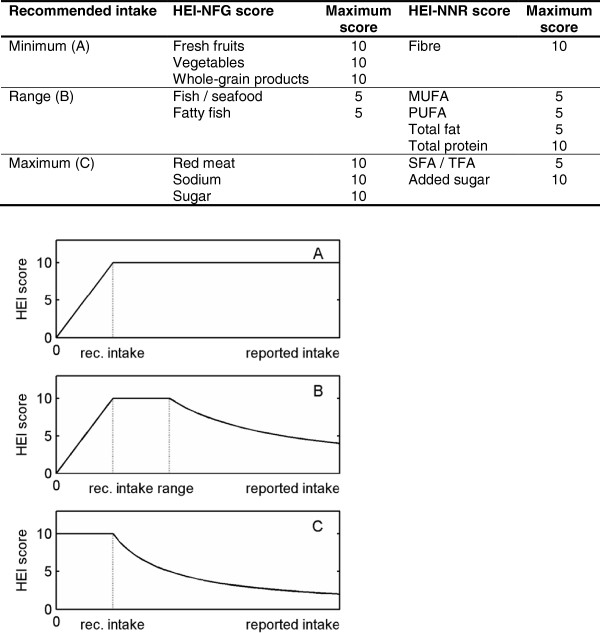
**Illustration of scoring algorithms of the HEI-NFG and HEI-NNR.** Abbreviations: HEI-NFG Healthy Eating Index based on Norwegian Food Guidelines, HEI-NNR Healthy Eating Index based on Nordic Nutrition Recommendations, rec recommended, MUFA Monounsaturated Fat, PUFA Polyunsaturated Fat, SFA/TFA Saturated Fat and Trans Fat.

When evaluating adherence to specific NFG recommendations, we further distinguished between food groups whose recommendations refers to (1) a minimum, (2) a range, or (3) a maximum of intake (see Figure 1), as described below, which is in line with the scoring algorithm of the German HEI [27].

(1) Food groups with a minimum of recommended intake (fruit, vegetables, and whole-grain) were scored directly from 0 to 10 according to equation 1.

(1) Score=reportedintakerecommendedintake×10

If intake exceeded the minimum recommended intake for fruits, vegetables, and whole-grain products, a maximum score of 10 was assigned.

(2) For food groups with a recommended intake range, both an intake below the lower recommended level as well as above the upper recommended level was evaluated by proportional score deduction. For intakes below the recommended lower intake level, equation 1 was used whereas equation 2 was applied for intakes above the upper recommended intake level.

(2) Score=recommendedintakereportedintake×10

The only food groups with a recommended range of intake were fish/seafood and fatty fish. Since ‘fatty fish’ was part of the variable ‘fish/seafood’ we divided each individual score by a factor 2 to avoid overestimation of fish in the total score. The maximum score for fish/seafood or fatty fish was therefore given by 5 instead of 10.

(3) Red meat, sodium,a and sugar are foods that should be consumed in limited amounts, i.e. the recommended intake refers to a maximum amount. Therefore, an intake below the recommendations was evaluated with a maximum score of 10 whereas overconsumption of such foods or nutrients was penalized by proportional score deduction according to equation 2. This also helps to consider the fact that overconsumption of such food items might easily lead to a positive energy balance and subsequent weight gain.

Finally, all component scores were summed to obtain a total score with a possible range from 0 to 70.

#### HEI-NNR score

Compliance with the Nordic Nutrition Recommendations (NNR) (see Table 1) was measured by means of a diet adherence index that is solely based on (macro-) nutrients, denoted as Healthy Eating Index (HEI)-NNR. Specifically, intake of the following macronutrients was considered: saturated and *trans* fatty acids (SFA + TFA), monounsaturated fatty acids (MUFA), polyunsaturated fatty acids (PUFA), total fat, added sugar, fibre, and total protein.

The HEI-NNR score was calculated based on the ratio of measured and recommended intake of the selected nutrients using an analogous scoring algorithm as for the HEI-NFG. That means that nutrients with a minimum of recommended intake (fibre), range of recommended intake (MUFA, PUFA, total fat, and total protein), or maximum values of recommended intake (SFA + TFA, added sugar) were calculated differently (see Figure 1). The possible score could range between 0 and 10 for added sugar, fibre, and total protein. Due to the large correlation between total fat and fat components, the scores given in equation 1 + 2 were multiplied by 0.5 in order to avoid an undue dominance of fat components in the total HEI-NNR score. Hence, for SFA + TFA, MUFA, PUFA, and total fat a maximum score of 5 was applied. Consequently, the total score could range between 0 and 50.

First, fibre represents a nutrient with a minimum of recommended intake. Therefore, an increasing intake was evaluated with a proportionally increasing score from 0 to 10 according equation 1. Intakes above the recommended minimum level were hence scored with the maximum score of 10.

Second, MUFA, PUFA, total fat as well as total protein are nutrients with a range of recommended intake, in which equation 1 was applied to score intakes below the recommendations whereas for intakes above the recommended range equation 2 was implemented. Intakes within the recommended range were evaluated with the respective maximum score, i.e. 5 for MUFA, PUFA, and total fat, respectively, and 10 for total protein.

Finally, SFA + TFA and added sugar represent nutrients that should be consumed in limited amounts. Consequently, intake below the recommended maximum were scored with the maximum of 5 (for SFA + TFA) or 10 points (for added sugar) while an excess of the recommended intake was penalised with proportional score deduction based on equation 2.

### Assessment of postpartum weight retention

Maternal weight development was assessed based on self-reports in questionnaires. Women were asked for their pre-pregnancy weight and height at study recruitment (week 15 of pregnancy). Total weight gain during pregnancy and postpartum weight of the women was assessed by a questionnaire that was administered 6 months after birth.

Postpartum weight retention was calculated as the difference of weight before and 6 months after pregnancy.

### Statistical analyses

The adherence to the Norwegian food-based guidelines (NFG) and Nordic Nutrition Recommendations (NNR) during pregnancy was measured by diet adherence indexes, namely the Healthy Eating Index (HEI)-NFG and the HEI-NNR.

The outcome variables were either weight retention 6 months after birth (difference between post- and pre-pregnancy weight in kg) or a binary variable describing risk of substantial postpartum weight retention (relative weight increase 6 months postpartum compared to pre-pregnancy weight ≥ 5% versus less). We used multiple linear and logistic regression analyses to examine the association between the continuous and the dichotomized outcome variable and each of the two HEI scores, respectively. The result was given in terms of the beta coefficient in units of kg per standard deviation (SD) of HEI-score (linear regression) or as odds ratio of substantial weight retention per SD of HEI-score (logistic regression).

All regression models were adjusted for the following covariates: maternal age (in years), maternal education (< 12 years, 12 years, 13-16 years, ≥ 17 years), household income (both incomes < 300,000 NOK, one income ≥ 300,000 NOK, both incomes ≥ 300,000 NOK), marital status (single or widow, married, cohabitants), pre-pregnancy BMI (in kg/m^2^), parity (primiparous, 1 previous pregnancy, 2 previous pregnancies, ≥ 3 previous pregnancies), weight of the child at birth (in grams), breastfeeding duration up to 6 months postpartum (in months), total energy intake estimated from the FFQ (in kcal/day), exercise during pregnancy (assessed in week 15: none, less than weekly, 1-2 times weekly, ≥ 3 times weekly), smoking during pregnancy (never, occasional, daily), and alcohol intake during pregnancy (assessed in week 22: never, < 0.5 times per week, ≥ 0.5 times per week).

Furthermore, we run a second model that was additionally adjusted for GWG (difference between weight before delivery and pre-pregnancy weight) as potential intermediate variable.

Differences between the HEI scores across categories of these covariates (e.g. age, education, etc.; see Table 2) were examined using Analysis of Variance for normally and Kruskal Wallis-Test for not-normally distributed variables.

**Table 2:** General characteristics of the MoBa sample (n = 47 011)

**Variable**	**Categories**	**N**	**%**	**HEI-NFG score**^**1**^	**HEI-NNR score**^**1**^
**Maternal age at delivery**	≤ 19 years	394	0.8	46.6 (7.7)	45.1 (3.2)
	20-24 years	4 884	10.4	47.4 (7.7)	45.7 (3.0)
	25-29 years	16 282	34.6	49.4 (7.3)	46.3 (2.6)
	30-34 years	17 679	37.6	50.4 (7.2)	46.6 (2.4)
	35-39 years	6 846	14.6	51.0 (7.1)	46.9 (2.2)
	≥ 40 years	926	2.0	52.0 (7.1)	46.9 (2.2)
**Pre-pregnancy BMI**	< 18.5 kg/m^2^	1 346	2.9	49.6 (7.6)	45.9 (2.7)
	18.5-24.9 kg/m^2^	31 012	66.0	50.2 (7.2)	46.4 (2.5)
	25-29.9 kg/m^2^	10 316	21.9	49.2 (7.4)	46.3 (2.6)
	30-34.9 kg/m^2^	3 195	6.8	48.7 (7.4)	46.3 (2.7)
	≥ 35 kg/m^2^	1 142	2.4	48.6 (7.4)	46.2 (2.8)
**Gestational weight gain**	< 10 kg	7 253	15.4	49.5 (7.5)	46.1 (2.8)
	10-15 kg	19 655	41.8	50.1 (7.2)	46.5 (2.5)
	16-19 kg	10 692	22.7	49.9 (7.2)	46.5 (2.5)
	≥ 20 kg	9 411	20.0	49.3 (7.4)	46.5 (2.5)
**Maternal education**	< 12 years	2 766	5.9	47.7 (7.9)	45.6 (3.1)
	12 years	11 488	24.4	48.4 (7.5)	45.9 (2.8)
	13-16 years	20 220	43.0	50.0 (7.1)	46.5 (2.4)
	17+ years	11 570	24.6	51.5 (6.9)	46.8 (2.2)
	Other/missing	967	2.1	49.4 (7.1)	46.1 (2.7)
**Household income**	Both incomes < 300 000 NOK	13 651	29.0	48.9 (7.4)	46.1 (2.7)
	One income ≥ 300 000 NOK	19 709	41.9	49.8 (7.3)	46.4 (2.5)
	Both incomes ≥ 300 000 NOK	12 476	26.5	50.9 (7.0)	46.7 (2.3)
	Missing data	1 175	2.5	49.4 (7.7)	46.0 (2.8)
**Marital status**	Married	21 675	46.1	50.4 (7.2)	46.6 (2.4)
	Single, widow	1 003	2.1	48.9 (7.9)	45.7 (3.1)
	Cohabitants	23 644	50.3	49.3 (7.4)	46.2 (2.6)
	Other or not known	689	1.5	49.6 (7.6)	45.9 (2.9)
**Parity**	Primiparous	25 797	54.9	49.7 (7.4)	46.3 (2.6)
	1 previous pregnancy	13 783	29.3	49.7 (7.3)	46.4 (2.5)
	2 previous pregnancies	6 020	12.8	50.5 (7.1)	46.6 (2.4)
	≥ 3 previous pregnancies	1 375	2.9	50.6 (7.4)	46.6 (2.4)
	Missing data	36	0.1	50.6 (8.2)	46.7 (2.3)
**Birth weight**	< 2 500 g	1 204	2.6	48.8 (7.6)	46.1 (2.7)
	≥ 2 500 g	45 807	97.4	49.8 (7.3)	46.4 (2.5)
**Breastfeeding duration (up to 6 months postpartum)**	None or < 1 month	1 519	3.2	48.0 (7.7)	45.9 (2.8)
	1-3 months	4 273	9.1	48.0 (7.6)	45.8 (2.9)
	4-5 months	2 600	5.5	48.8 (7.6)	46.0 (2.8)
	6 months	38 619	82.2	50.2 (7.2)	46.5 (2.4)
**Exercise during pregnancy (week 17)**	No	6 486	13.8	47.3 (7.5)	45.7 (3.0)
	Less than weekly	9 346	19.9	48.7 (7.0)	46.2 (2.6)
	1-2 times weekly	14 109	30.0	50.2 (7.1)	46.5 (2.4)
	≥ 3 times weekly	13 590	28.9	51.6 (7.1)	46.8 (2.3)
	Missing data	3 480	7.4	48.9 (7.6)	46.1 (2.7)
**Smoking during pregnancy**	No smoking during pregnancy	43 041	91.6	50.0 (7.2)	46.5 (2.5)
	Occasional	1 247	2.7	48.4 (7.8)	45.8 (2.8)
	Daily	2 420	5.2	46.4 (7.8)	45.2 (3.2)
	Missing	303	0.6	49.6 (7.7)	46.1 (3.0)
**Alcohol during pregnancy (week 22)**	Never	41 503	88.3	49.7 (7.3)	46.4 (2.6)
	< 0.5 times per week	4 588	9.8	50.5 (7.1)	46.5 (2.4)
	≥ 0.5 times per week	920	2.0	50.4 (7.1)	46.4 (2.5)

^1^Mean value (standard deviation).

The considered covariates were selected based on *a priori* knowledge and their observed association with the exposure and the outcome. Information on the covariates was obtained from questionnaires administered during pregnancy and after birth.

In a second set of analyses, we investigated the influence of the HEI-scores and covariates (except breastfeeding) on GWG as an intermediate outcome variable, either as continuous variable or dichotomized into excessive GWG (yes/no). Excessive GWG was defined depending on the pre-pregnancy BMI based on the updated IOM criteria [28], i.e. > 18 kg for BMI < 18.5 kg/m^2^, > 16 kg for BMI of 18.5-24.9 kg/m^2^, > 11.5 kg for BMI of 25-29.9 kg/m^2^, or > 9 kg for BMI ≥ 30 kg/m^2^, respectively.

Moreover, the impact of individual HEI-NFG and HEI-NNR components (per 1-point increment, each), on postpartum weight retention and GWG was studied using analogous linear regression models.

All analyses were performed with SAS software, version 9.3 (SAS Institute Inc., Cary, NC, USA).

## Results

Women in the present MoBa sample had an average age of 30.0 ± 4.6 years at baseline and a mean pre-pregnancy BMI of 24.0 ± 4.2 kg/m^2^. The participants gained on average 15.0 ± 6.0 kg during pregnancy and their mean weight retention 6 months after birth amounted to 1.2 ± 4.8 kg (data not tabulated).

The majority of women were rather highly educated (13+ years) and primiparous. Nearly 60% were physically active during the pregnancy on at least a weekly basis. As expected, the proportion of women who reported regular smoking or alcohol consumption during their pregnancy was very small (see Table 2). The mean HEI-NFG score amounted to 49.8 ± 7.3 and the mean HEI-NNR score was 46.4 ± 2.5. Both HEI scores increased slightly with increasing age, socio-economic status, exercise frequency, and breastfeeding duration, whereas the HEI scores tended to decrease with increasing pre-pregnancy BMI, GWG, and smoking frequency (Table 2). All HEI score differences across categories were statistically significant (p value < 0.0001), except for the HEI-NNR score across categories of alcohol intake.

Concerning intake of food groups considered in the NFG, the participants showed good adherence to the recommendations for whole-grain, red meat and added sugar intake, whereas the adherence to recommended vegetable and fatty fish intake was low (see Table 3, upper part). Interestingly, concerning nutrient intake, there was a high proportion of women showing compliance with the recommendations for nearly all macronutrients, except of saturated fat (Table 3, lower part).

**Table 3:** Dietary intake and adherence to Nordic dietary guidelines (adherence scores and % of adherence)

**Food groups/Macronutrients**	**Mean (STD)**	**Adherence score: Mean (STD)**	**% of women adhering to recommendations**
***Food groups***		***HEI-NFG***	
**Fresh fruits (g/day)**	263.7 (191.7)	6.9 (3.0)	32.9
**Vegetables (g/day)**	151.6 (90.0)	4.9 (2.5)	6.5
**Whole-grain (g/day)**	116.7 (97.3)	7.4 (3.7)	61.1
**Fish and seafood (g/week)**	259.0 (157.9)	3.5 (1.4)†	22.8
**Fatty fish (g/week)**	75.2 (92.3)	1.6 (1.5)†	6.7
**Red meat (g/week)**	527.5 (195.6)	8.8 (1.5)	44.7
**Sodium (g/day)**	3.1 (0.8)	8.0 (1.5)	19.4
**Added sugar (E%)**	10.7 (5.0)	8.7 (1.8)	50.9
***Macronutrients***		***HEI-NNR***	
**Total fat (E%)**	31.1 (4.5)	4.9 (0.2)^†^	72.5
**Saturated +** ***trans *** **fat (E%)**	12.9 (2.2)	4.0 (0.6)^†^	8.2
**Monounsaturated fat (E%)**	10.0 (1.8)	4.6 (0.5)^†^	47.1
**Polyunsaturated fat (E%)**	5.9 (1.6)	4.8 (0.4)^†^	65.4
**Fibre (g/day)**	30.8 (10.4)	9.4 (1.2)	69.6
**Added sugar (E%)**	10.7 (5.0)	8.7 (1.8)	50.9
**Total protein (E%)**	15.3 (2.1)	10.0 (0.1)	97.7
***Total energy intake (kcal/day)***	2306.7 (606.8)		

*Abbreviations*: *E%* percentage of total energy intake, *HEI* Healthy Eating Index, *NFG* Norwegian Food Guidelines, *NNR* Nordic Nutrition Recommendations, *STD* Standard Deviation.

^†^Maximum score was 5 whereas for the rest of the components the maximum score was 10.

A higher HEI-NFG score (reflecting higher overall adherence to the NFG) was inversely associated with weight retention 6 months after birth. In contrast, a higher HEI-NNR score, which quantifies increasing compliance with the NNR, was not related to weight retention 6 months after birth (see Table 4). However, after additional adjustment for GWG as a potential intermediate, the association of the HEI-NFG score with postpartum weight retention was attenuated towards a marginal inverse relationship. On the contrary, the association of the HEI-NNR score with postpartum weight retention became inverse upon adjustment for GWG. These inverse associations of the HEI-NFG and HEI-NNR with weight retention after birth were independent of total energy intake.

**Table 4:** Adherence to Norwegian food guidelines or NNR (measured by the HEI) and postpartum weight retention

	**β-coefficient**^**1**^	**SE**	***p *** **value**	**Adjusted R**^**2 **^**of the model**
**Postpartum weight retention**^**2**^				
HEI-NFG per standard deviation	-0.058	0.024	0.017	0.058
HEI-NNR per standard deviation	0.014	0.024	0.570	0.058
**Postpartum weight retention**^**2**^**, additionally adjusted for gestational weight gain (GWG)**				
HEI-NFG per standard deviation	-0.034	0.020	0.094	0.314
HEI-NNR per standard deviation	-0.074	0.020	0.0003	0.315
**GWG as intermediate outcome**^**3**^				
HEI-NFG per standard deviation	-0.062	0.029	0.033	0.115
HEI-NNR per standard deviation	0.196	0.029	< 0.0001	0.116

*Abbreviations*: *HEI* Healthy Eating Index, *NFG* Norwegian Food Guidelines, *NNR* Nordic Nutrition Recommendations, *SE* Standard Error.

^1^Units = kg per HEI-score standard deviation ( = 7.31 for HEI-NFG, 2.54 for HEI-NNR).

^2^Linear regression model, adjusted for maternal age, maternal education, household income, marital status, pre-pregnancy BMI, parity, weight of the child at birth, breastfeeding duration up to 6 months postpartum, total energy intake (kcal), exercise during pregnancy (week 15), smoking during pregnancy, alcohol intake during pregnancy (week 22).

^3^Analogous model but without adjustment for breastfeeding duration.

When studying the role of GWG as an intermediate outcome, it turned out that the HEI-NFG score was inversely associated with GWG while the HEI-NNR score was rather strongly directly related with GWG (Table 4). In a sensitivity analysis where excessive GWG was studied as the outcome, the same direction of associations of the HEI-NFG and HEI-NNR was observed (data not shown).

Table 5 represents the analyses of single HEI-NFG or HEI-NNR components, being mutually adjusted, respectively, in relation to weight retention 6 months after birth or GWG (both modelled as continuous variables). Among the single HEI-NFG score components only higher intake of fish was associated with less postpartum weight retention, which was also observed when adjusted for GWG.

**Table 5:** Association of single HEI component scores with postpartum weight retention and gestational weight gain (GWG)

**Score component**	**Higher score reflects**	**Postpartum weight retention**^**1**^			**Postpartum weight retention**^**1**^**, + adjustment for GWG**			**GWG as intermediate outcome**^**2**^		
		**β**^**3**^	**SE**	***p *** **value**	**β**^**3**^	**SE**	***p *** **value**	**β**^**3**^	**SE**	***p *** **value**
***HEI-NFG***										
Fresh fruits	Higher intake	0.0003	0.009	0.969	0.003	0.008	0.718	-0.007	0.011	0.486
Vegetables	Higher intake	0.004	0.011	0.711	-0.001	0.009	0.913	0.010	0.013	0.418
Whole-grain	Higher intake	-0.006	0.006	0.340	-0.001	0.005	0.796	-0.011	0.008	0.147
Fish	Higher adherence to recommendations^4^	-0.067	0.020	0.001	-0.051	0.017	0.003	-0.039	0.024	0.109
Fatty fish	Higher adherence to recommendations^4^	-0.002	0.018	0.913	0.019	0.015	0.209	-0.049	0.022	0.024
Red meat	Lower intake	-0.029	0.018	0.104	-0.013	0.015	0.402	-0.039	0.022	0.074
Sodium	Lower intake	-0.025	0.030	0.409	0.044	0.026	0.091	-0.160	0.037	< 0.0001
Added sugar	Lower intake	0.022	0.016	0.172	-0.018	0.014	0.207	0.091	0.020	< 0.0001
***HEI-NNR***										
Total fat	Higher adherence to recommendations^4^	-0.015	0.110	0.890	-0.196	0.094	0.038	0.410	0.134	0.002
SFA + TFA	Lower intake	-0.111	0.053	0.034	0.019	0.045	0.670	-0.303	0.064	< 0.0001
MUFA	Higher adherence to recommendations^4^	0.056	0.069	0.417	0.138	0.059	0.020	-0.197	0.084	0.019
PUFA	Higher adherence to recommendations^4^	0.017	0.063	0.788	-0.066	0.053	0.218	0.201	0.076	0.008
Fibre	Higher intake	-0.009	0.025	0.708	-0.037	0.021	0.083	0.058	0.030	0.053
Added sugar	Lower intake	0.020	0.015	0.170	-0.025	0.013	0.044	0.104	0.018	< 0.0001
Total protein	Higher adherence to recommendations^4^	-0.112	0.202	0.578	-0.045	0.172	0.794	-0.168	0.244	0.491

*Abbreviations*: *HEI* Healthy Eating Index, *MUFA* Monounsaturated Fat, *NFG* Norwegian Food Guidelines, *NNR* Nordic Nutrition Recommendations, *PUFA* Polyunsaturated Fat, *SE* Standard Error, *SFA + TFA* Saturated and *Trans* Fat.

^1^**Score components were mutually adjusted** within a linear regression model, that was further adjusted for maternal age, maternal education, household income, marital status, pre-pregnancy BMI, parity, weight of the child at birth, breastfeeding duration up to 6 months postpartum, total energy intake, exercise during pregnancy (week 15), smoking during pregnancy, alcohol intake during pregnancy (week 22).

^2^Analogous model but without adjustment for breastfeeding duration.

^3^β-coefficient denotes change in weight retention at 6 months postpartum (in kg) per 1-point-increment of each HEI component score.

^4^Too low as well as too high intake results in a deduction of points.

Concerning individual HEI-NNR components, a lower intake of saturated and *trans* fat (SFA + TFA) was significantly associated with reduced postpartum weight retention. After additional adjustment for GWG, however, no association remained indicating that the initial observed association of a lower SFA + TFA intake with reduced postpartum weight retention is explained by its association with reduced GWG.

Instead, after additional adjustment for GWG, a higher adherence to recommended fat intake and a lower intake of added sugar appeared to be associated with decreased postpartum weight retention, as a consequence of their positive association with GWG. On the other hand, a higher adherence to recommended intake of monounsaturated fatty acids (MUFA) was directly associated with postpartum weight after additional adjustment for GWG, while it was inversely related to GWG as outcome variable. These results, however, should be interpreted with some caution as adjusting for a mediator variable is critical, particularly if the association of the exposure with the outcome (postpartum weight retention) point in a different direction than its association with the intermediate (GWG).

**Figure 2:**
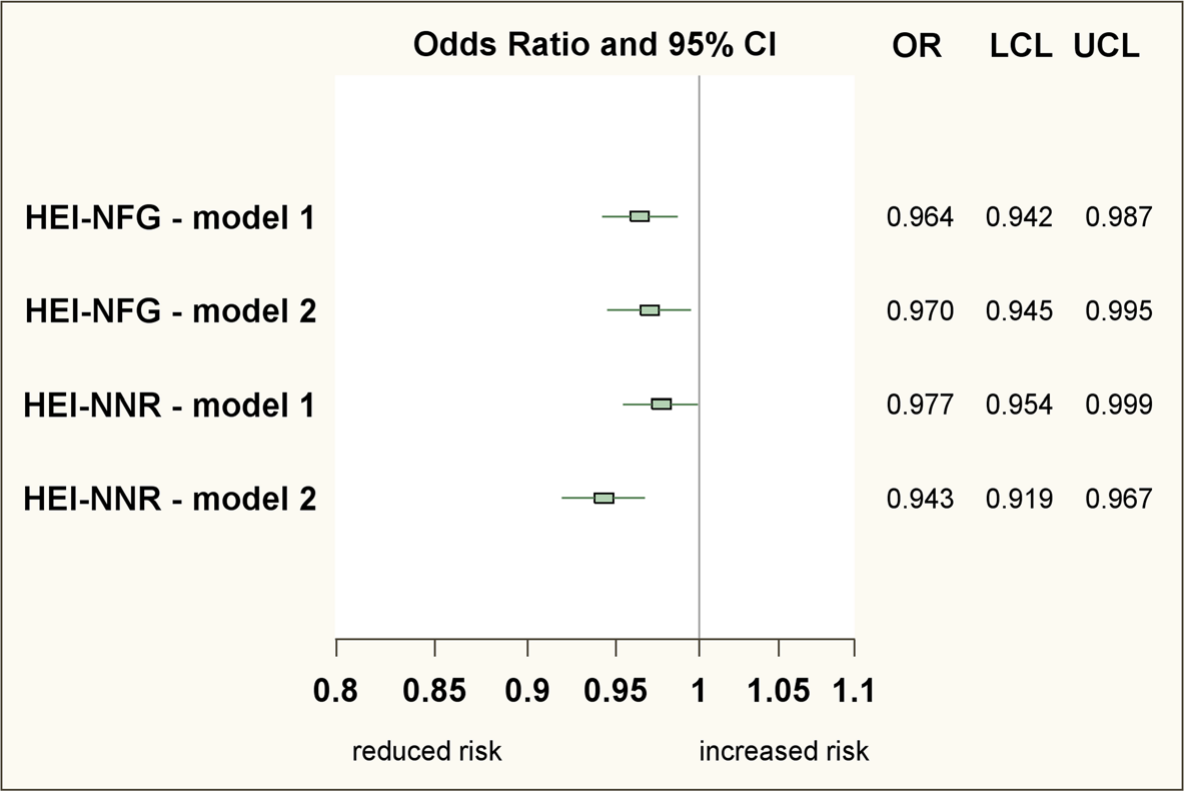
**Adherence to Norwegian food guidelines or NNR and risk of substantial postpartum weight retention.** Abbreviations: CI Confidence Interval, HEI Healthy Eating Index, LCL Lower Confidence Limit, NFG Norwegian Food Guidelines, NNR Nordic Nutrition Recommendations, OR Odds ratio, UCL Upper Confidence Limit. Substantial postpartum weight retention is defined as having gained at least 5% to the pre-pregnancy weight. Odds ratios of having substantial postpartum weight retention are modelled per standard deviation-increment of each HEI score. ***Logistic regression model 1:*** adjusted for maternal age, maternal education, household income, marital status, pre-pregnancy BMI, parity, weight of the child at birth, breastfeeding duration up to 6 months postpartum, total energy intake, exercise, smoking, and alcohol intake during pregnancy. ***Logistic regression model 2***: additionally adjusted for gestational weight gain.

Finally, we investigated the role of adhering to either the NFG or the NNR for preventing substantial weight retention 6 months after birth, which was defined on the relative scale (i.e. ≥ 5% of weight gain compared to pre-pregnancy weight). According to this definition, 28% of the cohort had experienced substantial postpartum weight retention. The associations of the corresponding diet adherence scores, namely the HEI-NFG and HEI-NNR, with substantial postpartum weight retention are illustrated in Figure 2. Both the HEI-NFG and the HEI-NNR were inversely related to risk of substantial weight retention 6 months after birth (Odds Ratio (OR) and 95% Confidence Intervals (CI) per 1 STD-increment: 0.96 (0.94-0.99) for the HEI-NFG and 0.98 (0.95-1.00) for the HEI-NNR). Also after additional adjustment for GWG, these inverse associations were stable.

## Discussion

In this analysis using the unique MoBa data, adherence to official Norwegian food guidelines (as measured by the HEI-NFG) was associated with reduced weight retention 6 months after birth. After inclusion of GWG as potential mediator into the linear regression models, the inverse association of the HEI-NFG was slightly weakened. This indicates that this association can be partly explained by the inverse association between HEI-NFG and GWG. In contrast, we found that a higher compliance with the HEI-NNR was associated with a higher GWG, while it was not predicting postpartum weight retention as continuous variable. When adjusting for GWG, a strong inverse relationship between HEI-NFG and postpartum weight retention was seen. These divergences concerning the observed associations of the HEI-NFG and HEI-NNR particularly with GWG can be explained by the fact that the respective underlying dietary recommendations measure different constructs. This is also reflected by the rather low correlation between the HEI-NFG and HEI-NNR (Spearman’s r: 0.41). In addition, the HEI-NFG components are generally independent of each other whereas some HEI-NNR components (particularly total fat and fat components) are highly correlated, which makes the HEI-NNR more difficult to interpret. Nevertheless, the associations of the HEI-NFG and HEI-NNR were in the same direction, i.e. inverse, when studying risk of substantial postpartum weight retention as the outcome variable, independent of adjustment for GWG (Figure 2). Notably, these inverse associations of the HEI-NFG and HEI-NNR with weight retention after birth appeared to be independent of total energy intake, implying that not only total diet quantity but also diet quality plays a role for weight management. There are also some previous studies that suggested a protective role of adhering to specific dietary guidelines for subsequent weight gain, independent of total caloric intake, in white adult cohort populations [29-31]. However, we are not aware of comparable studies being conducted within the specific population group of pregnant women.

Surprisingly, only a few of the individual components of the NFG and NNR were significantly related to reduced postpartum weigh retention in the mutually adjusted model, namely fish and seafood within the NFG, and saturated + *trans* fat in case of the NNR (model without adjustment for GWG). This can be explained by the fact that the components were mutually adjusted for each diet score, indicating that a dietary pattern as a whole has different health effects than single dietary components. For instance, we could observe that a lower red meat intake was inversely related to postpartum weight if entered individually to the model (data not shown). This phenomenon, however, might be explained by a simultaneous increase in intake of other food groups, like fish for instance, as this association was reduced after mutual adjustment for other relevant food groups.

A direct comparison of our results with that from previous research is difficult since the majority of such studies had different research questions, e.g. focussing only on the effect of selected dietary components during pregnancy on maternal weight development after pregnancy instead of studying dietary patterns. Martins *et al*. [14], for instance, found that an increased intake of saturated fat or processed foods during pregnancy was connected to an increment in postpartum weight retention. However, this analysis considered only a few covariates. In fact, these associations were mainly explained by an increased total energy intake, as the observed effect of saturated fat and processed foods was eliminated after further adjustment for energy intake. Other studies only considered the postpartum period, thereby disregarding the effect of diet during pregnancy. Such studies found that an increased intake of *trans* fat [32] or junk food (i.e. sweetened beverages, French fries and chips, fast food) [33] after birth is associated with increased postpartum weight retention. In addition, there is one recent longitudinal study showing that diet quality postpartum, assessed by the adherence to either a Mediterranean style diet or the Dietary guidelines for Americans, was not associated with postpartum weight retention [34]. In contrast, Stendell-Hollis *et al.* found in an interventional trial that adherence to either a Mediterranean style diet or the US Department of Agriculture (USDA) My Pyramid diet supported the promotion of postpartum weight loss [35] but this study only covers the lactation period. Hence, it is not clear whether this is transferable to the effect of diet during pregnancy on postpartum weight.

Major strengths of the present study include the prospective cohort design and the large sample size, which ensures large variation in dietary habits. In addition, the use of diet adherence scores, namely the HEI-NFG and HEI-NNR, enabled us to capture the overall compliance to official Norwegian food-based guidelines or the nutrient-based Nordic Nutrition Recommendations, respectively. Thus, these dietary adherence scores are able to reflect complex dietary patterns instead of just single aspects of the diet.

Despite the high number of participants, the MoBa sample is not truly representative of all pregnant Norwegian women since only 40.6% of those pregnant women who are invited to this study agreed to participate. A comparison with non-participating pregnant Norwegian women showed that participating women comprise less young mothers (<25 years) and less women living alone as well as less women with two or more previous pregnancies [36]. Nevertheless, a study of potential self-selection bias showed that despite a different prevalence of exposures and outcomes compared to the total population of pregnant women, no statistically relevant differences regarding eight selected exposure-outcome associations were found [36].

A further study limitation is the lack of data on maternal diet in the postpartum period. Still, we have data on important lifestyle variables (namely smoking, alcohol intake, and physical activity) from the postpartum period and further adjustment for postpartum lifestyle factors did not markedly affect our observed associations. Concerning maternal diet, we assume that some aspects of eating during pregnancy are likely to continue during the postpartum period, as also reported in the literature [37], and that both pre- and postpartum diets are contributing factors to postpartum weight retention.

A further issue of concern is that the considered postpartum period of 6 months may be a too short period to explore weight retention since 82.2% of women declared breastfeeding up to 6 months postpartum. Therefore, a subgroup analysis among women with breastfeeding duration < 6 months postpartum was conducted. In doing so, it turned out that the observed association between increased adherence to Norwegian food guidelines and decreased postpartum weight retention was still evident and even strengthened. Conversely, the relationship between compliance to Nordic Nutrition Recommendations and postpartum weight retention was weakened (data not shown)*,* which can be explained by the smaller variability of the HEI-NNR compared to the HEI-NFG score.

In addition, the assessment of the diet using FFQs bears the risk that either conscious or unconscious misreporting errors occur. It has been shown that the intake of foods which are perceived as “unhealthy” tends to be under-reported in women with higher BMI [38]. Another challenge is that it is particularly difficult for the participants to assess correct intakes of foods in early pregnancy when many women are suffering from nausea or experiencing changes in appetite and eating patterns. However, the MoBa FFQ was developed especially for the use in pregnancy and a previous study comparing its estimates with those from a 4-day food diary as well as biological markers showed satisfactory results concerning its validity [18]. Finally, also body weight was assessed based on self-reports and is therefore vulnerable to reporting error, which has been described in detail previously [39].

## Conclusion

To our knowledge, this is the first study to examine whether adherence to dietary guidelines during pregnancy is associated with postpartum weight retention. We found that even after adjustment for several potential confounders, a higher adherence to the official Norwegian food guidelines was related to reduced weight retention 6 months after birth in all models. The Nordic Nutrition Recommendations, however, are not associated with postpartum weight retention as continuous outcome but inversely associated with the categorical outcome (risk of substantial postpartum weight retention). Although these associations appeared to be small in magnitude, they were statistically significant, suggesting a role of complying with official national dietary recommendations for preventing postpartum weight retention, thereby reducing the risk of adverse future weight development in pregnant women. Thus, promoting knowledge on national dietary guidelines among pregnant women may help not only to ensure an adequate nutrient supply for the mother and the unborn child, but also to contribute to prevention of undesirable maternal weight development. From our data, adherence to food-based dietary guidelines appears to have a more important role for prevention of postpartum weight retention compared with nutrient-based dietary recommendations.

However, there is still a need for more prospective cohort studies addressing this research topic, with further consideration of postpartum changes in diet, and/or interventional trials that investigate effects of specific dietary patterns during pregnancy on maternal weight development during and after pregnancy. This may help to potentially confirm the role of national dietary guidelines with regard to successful weight management in the critical time window of pregnancy and the postpartum period.

## Abbreviations

BMI: Body mass index; FFQ: Food frequency questionnaire; GWG: Gestational weight gain; HEI: Healthy eating index; NFG: Norwegian food-based guidelines; NNR: Nordic Nutrition Recommendations; MoBa: The Norwegian Mother and Child Cohort Study; MUFA: Monounsaturated fatty acids; PUFA: Polyunsaturated fatty acids; SFA: Saturated fatty acids; TFA: *Trans* fatty acids.

## Competing interests

The authors declare that they have no competing interests.

## Authors’ contributions

AvR analysed the data and wrote the manuscript, LL was responsible for conception of the analysis, ALB, MH and HMM contributed to acquisition of the data and study design, all authors contributed to interpretation of the data, critical review and final approval of the manuscript.

## Pre-publication history

The pre-publication history for this paper can be accessed here:

http://www.biomedcentral.com/1471-2458/14/75/prepub

## Supplementary Material

Additional file 1: Table S1 Definition of food groups considered within the Norwegian Food Guidelines. Click here for file
