# Supplementary material for: Adherence of pregnant women to Nordic dietary guidelines in relation to postpartum weight retention: results from the Norwegian Mother and Child Cohort Study
Source: BMC Public Health. 2014 Jan 24;14:75. doi: 10.1186/1471-2458-14-75 (PMC3908932; doi:10.1186/1471-2458-14-75)
Supplement: Additional file 1: Table S1 — Definition of food groups considered within the Norwegian Food Guidelines. [file 1471-2458-14-75-S1.doc]

**Additional file 1: Table S**1: Definition of food groups considered within the Norwegian Food Guidelines

| **Food group** | **Assigned FFQ items / variables** |
| --- | --- |
| Fresh fruits | Oranges, Bananas, Grapes, Apples, Peach, Grapefruit, Strawberries, Other berries, Mango, Melon, Papaya, Plum, Pear, Other fruits |
| Vegetables | Frozen vegetables, Cucumber, Aubergine, Avocado, Cauliflower raw, Cauliflower cooked, Broccoli raw, Broccoli cooked, Green beans, Peas, Carrots raw, Carrots boiled, Cabbage raw, Cabbage cooked, Garlic, Swede  raw, Swede boiled, Onions raw, Onions cooked, Corn on cob , Pepper raw, Peppers casserole, Brussels sprouts, Green mixed salad, Lettuce, Celery, Mushrooms raw, Mushroom fried casseroles, Mushroom, Spinach, Squash , Tomato, Other vegetables |
| Whole-grain | Bread whole meal, Bread fibre, Muesli unsweetened, Porridge from oat |
| Fish | Cod, Mackerel, Salmon, Halibut, Tuna, Other fishes, Fish burger, Fish fingers/breaded fish, Fish casserole, Shrimps, Mussels, Crab, Sardines, Sardines in oil, Smoked salmon, Herring pickled, Shrimps northern, Crab canned, Tuna spread, Other fish spread |
| Fatty fish | Mackerel, Salmon, Sardines, Sardines oil, Smoked salmon, Herring pickled |
| Red meat | Sausage with beef and/or pork, Hot dogs, Meatballs, Hamburger, Minced meat, Beef roast, Beef, T-bone steak, Beef stew, Pork chops, Pork tenderloin, Pork loin smoked, Spareribs, Bacon, Pork stew, Lamb roast, Lamb stews, Reindeer roast, Roast elk, Reindeer patty, Stew game, Game |
| Salt | Sodium contributed by food |
| Added sugar | Added sugar, Sugar added to tea and coffee |
